# Generation, culture, and stimulation of small intestinal murine organoids in parasitology research

**DOI:** 10.1016/j.xpro.2023.102608

**Published:** 2023-09-25

**Authors:** Marta Campillo Poveda, Claire Drurey, Rick M. Maizels

**Affiliations:** 1 Wellcome Centre of Integrative Parasitology, School of Infection and Immunity, University of Glasgow, Glasgow, UK

**Keywords:** Flow Cytometry/Mass Cytometry, Immunology, Microscopy, Gene Expression, Organoids

## Abstract

Parasitic helminth worms frequently infect the gastrointestinal tract and interact with the intestinal epithelium and specialized cell types within it. Intestinal organoids derived from stem cells that line the intestine represent a transformational technology in the study of epithelial-parasite dialogue. Here, we present a protocol for establishing small intestine organoid cultures and administering parasite products of interest to these cultures. We then describe steps for evaluating their impact by microscopy, flow cytometry, immunohistology, and mRNA gene expression.

For complete details on the use and execution of this protocol, please refer to Drurey et al. (2022).^1^

## Highlights

• Establish organoids derived from stem cells found in crypts lining the intestine
• Stimulate organoid cultures with parasite products of interest
• Assess impact by microscopy, flow cytometry, immunohistology, and mRNA gene expression

## Abstract (editor highlights)

Publisher’s note: Undertaking any experimental protocol requires adherence to local institutional guidelines for laboratory safety and ethics.

## Graphical abstract

**Figure:**
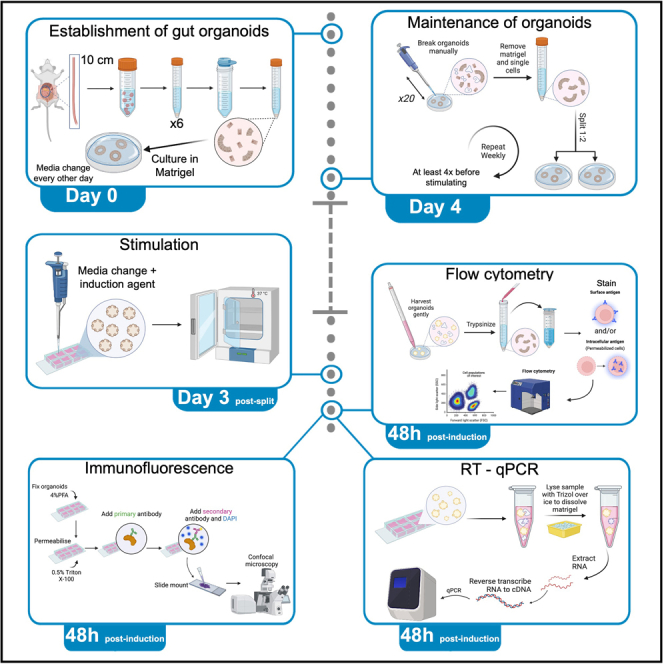

## Before you begin

This paper describes the generation, culture, and stimulation of small intestinal murine organoids as a model for parasitology research with a particular focus on helminth (worm) parasites which inhabit the intestinal niche such as *Heligmosomoides polygyrus*. The organoids are generated from small intestinal crypts of C57BL/6 mice and cultured in a three-dimensional matrix.^2^^,^^3^ The resulting organoids displayed characteristic intestinal epithelial cell markers and could be differentiated into various intestinal cell types under the influence of key cytokines. To investigate the response of these organoids to parasitic infection, they can also be stimulated with parasite products, or even co-cultured with live parasite organisms. Organoids are an important tool to study epithelial changes during parasitic infections *in vitro*. Our lab, amongst others, found that stimulating organoids with parasitic products alters the differentiation program of intestinal stem cells, mirroring some of the changes observed *in vivo*.^1^^,^^4^ These findings suggest that small intestinal murine organoids provide a valuable model for studying host-parasite interactions in parasitology research.

Make sure all the materials are stored according to manufacturer’s conditions.

## Key resources table

**Table:** 

REAGENT or RESOURCE	SOURCE	IDENTIFIER
**Antibodies**		

Anti-DCAMKL1 antibody	Abcam	ab31704
Goat anti-rabbit IgG (H + L) secondary antibody, FITC	Invitrogen	65-6111
Fixable Viability Dye eFluor 506	eBioscience	65-0866-18
Brilliant Violet 605 anti-mouse Ki-67	BioLegend	652413
CD326 (EpCAM) monoclonal antibody (G8.8), biotin	eBioscience	13-5791-80
Anti-cytokeratin, pan antibody, mouse monoclonal	Sigma-Aldrich	C5992
*Ulex europaeus* (gorse) agglutinin I (UEA I), fluorescein (FITC)	Invitrogen	L32476
PE/cyanine7 anti-mouse CD24 antibody	BioLegend	101821
DAPI (1:10,000)	Invitrogen	D1306

**Other**		

Matrigel (MG)	R&D	356231
EGF	Invitrogen	PMG8044
Noggin	PeproTech	250-38
R-spondin	R&D	3474-RS-050
CHIR99021	Stemgent	04-0004
B-27 supplement (50×), minus vitamin A	Invitrogen	12587-010
N-2 supplement (100×)	Invitrogen	17502-048
PBS	Gibco	14190094
EDTA	Invitrogen	15575020
Advanced DMEM/F12 medium	Invitrogen	12634-028
HEPES	Gibco	15630056
L-glutamine	Gibco	25030024
Penicillin/Streptomycin (Pen/Strep)	Gibco	15140122
Cell Recovery freezing media	Gibco	12648010
50 mL Falcon tube	Any supplier	NA
15 mL Falcon tube	Any supplier	NA
Petri dish	Any supplier	NA
Serological pipette	Any supplier	NA
70 μm cell strainer	Greiner	542070
20 μm cell strainer	Greiner	542120
24-well plate	Corning	3524
Coverslips	Smith Scientific	NPS13/2222
Nunc Lab-Tek II Chamber Slide System – 8-well chamber slide	Thermo Fisher Scientific	154534
DNase I, grade II	Sigma-Aldrich	10104159001
Glycine	Fisher Chemical	10101620
TrypLE Express Enzyme	Gibco	12605010
FCS, qualified, HI	Gibco	10500-064
Bovine serum albumin (BSA)	Invitrogen	15561020
Sodium azide (NaAZ)	Thermo Scientific Chemicals	014314.22

## Materials and equipment

### Dissection media

PBS(1 x): 20 mL per gut - Store at 4°C for up to 1 month.

**Table:** Advanced DMEM/F12 base (ADF Base)

Reagent	Final concentration	Amount
Advanced DMEM/F12	1×	500 mL
HEPES 1 M	10 mM	5 mL
Pen/Strep 10000 U/mL / 10 mg/mL	100 U/mL / 100 μg/mL	5 mL
Glutamine 200 mM	2 mM	5 mL

Store at 4°C for up to 1 month.

**Table:** Advanced DMEM/F12 Organoids – 50 mL (ADF Org)

Reagent	Final concentration	Amount/Notes
ADF Base	1×	48 mL
N2 supplement	1×	0.5 mL
B27 supplement	1×	1 mL

Store at 4°C for up to 1 month.

**Table:** Culture media - ADF Org + supplements – well number dependent (0.5 mL/well)

Reagent	Final concentration	Amount/Notes	For 10 wells media change
ADF org	1×	Well number dependent	4.85 mL
EGF	50 ng/mL	Well number dependent	50 μL
Noggin	100 ng/mL	Well number dependent	50 μL
R-spondin	500 ng/mL	Well number dependent	50 μL
CHIR99021	3 μM	Only for initial plating	-

Store at 4°C for up to 1 month.

**Table:** FACS buffer

Reagent	Final concentration	Amount/Notes
PBS	1×	500 mL
BSA	0.5%	2.5 mL
NaAz	0.05%	250 μL

Store at 4°C for up to 1 month.

**Table:** Immunofluorescence (IF) Buffer

Reagent	Final concentration	Amount/Notes
PBS	1×	90 mL
Triton X-100	0.1%	100 μL
Tween 20	0.05%	50 μL
FCS	10%	10 mL

Store at 4°C for up to 2 months.

**Table:** Antibody Dilution Buffer

Reagent	Final concentration	Amount/Notes
PBS	1×	40 mL
BSA	1%	400 μL
Triton X-100	0.1%	40 μL

Store at 4°C for up to 2 months.

## Step-by-step method details

### Crypt isolation and establishment of gut organoids

**Timing: Days 0–6**

**Figure 1:**
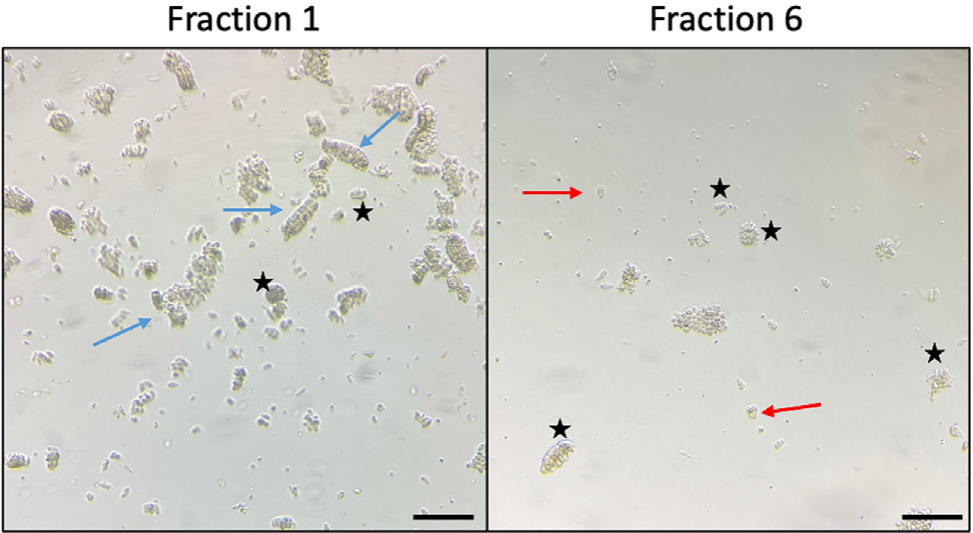
Images of the different fractions under 4× objective Black stars represent intestinal crypts; blue arrows represent villi and other big debris; red arrows represent single cells.

**Figure 2:**
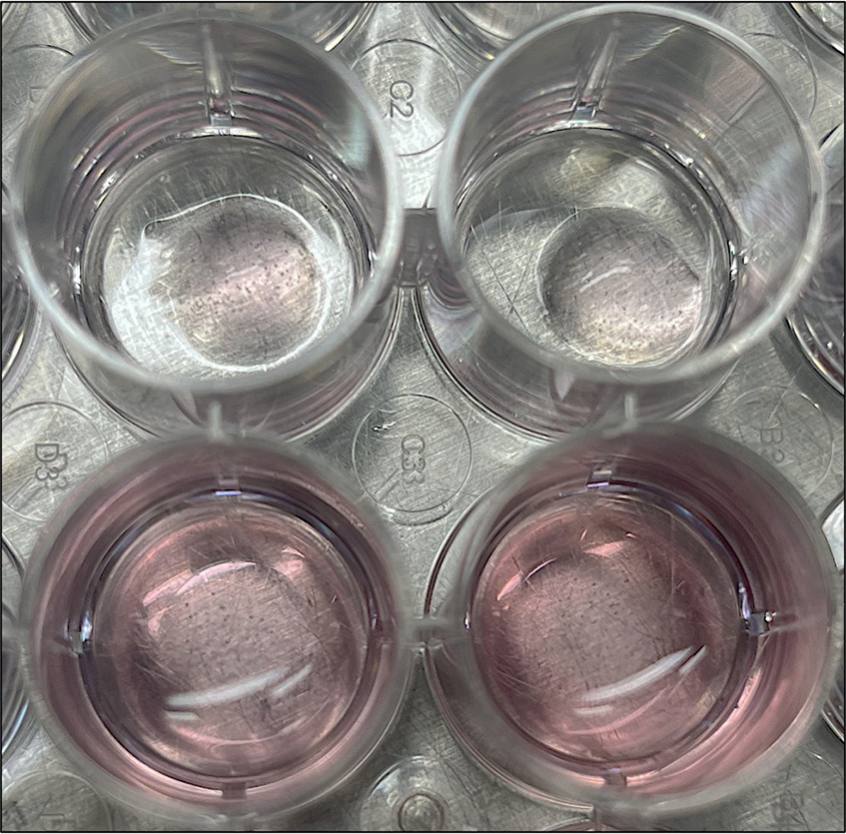
Example of MG domes, Top: prior to media addition; bottom: with 500 μL/well of ADF org media

1. Tissue collection - In the animal facility (10–15 min).
  a. Dissect the most proximal 10 cm of the small intestine, the zone inhabited by *H. polygyrus,* and flush with 5 mL ice cold PBS using a 5 mL syringe and a 200 μL pipette tip. Cut longitudinally and wash out the remaining intestinal contents. Scrape GENTLY with a coverslip to remove upper mucosa.
  b. Transfer to a 50 mL Falcon tube with 15 mL ice-cold PBS.
  c. Transport back to the laboratory on ice.
2. Crypt isolation - Back in the lab, work inside a hood.
  a. Transfer the Falcon tube contents onto a Petri dish, and use scissors to cut gut tissue into small pieces (1–2 mm).
  b. Transfer to a fresh 50 mL Falcon tube with a 10 mL serological pipette and make up to a total volume of 10 mL with ice-cold PBS.
  c. Pipette up/down with a 10 mL serological pipette GENTLY ×10. Allow settling.
  d. Remove PBS and repeat wash with 10 mL ice-cold PBS twice more.
  e. Aspirate PBS and add 20 mL 2 mM EDTA in ice-cold PBS and incubate for 30 min at 4°C on a roller.
  f. Allow tissue fragments to settle, aspirate the PBS-EDTA and add 10 mL ice-cold PBS and mix GENTLY ×10.
  g. Allow tissue fragments to settle, collect supernatant in a 15 mL tube (this is FRACTION 1).
  h. Repeat (with more vigorous pipetting each time) until you have 6 fractions.
  i. Transfer 20 μL from each fraction into a 96-well plate to look at the crypt concentration under a 4× objective in an inverted microscope. Take the crypt-enriched fractions for culture.
    Tip: Avoid fractions with a lot of single cells or villi - typically take fractions 2–6 (Figure 1).
  j. Combine crypt-enriched fractions into a 50 mL Falcon tube through a 70 μm cell strainer.
  k. Wash the cell strainer with 5 mL ice-cold PBS and spin at 300 *g* for 3 min at 4°C to pellet crypts. Use a swing-out rotor rather than an angled rotor to sediment the crypts,
    Tip: If crypts fail to pellet add ice cold PBS (∼10 mL) and repeat at 400 *g* to help spin down. A swinging bucket centrifuge is needed to pellet the crypts at the bottom of the tubes.
  l. Thaw MG on ice to prevent solidifying. Alternatively, thaw MG at 4°C before starting and keep on ice to prevent solidifying.
  m. Remove supernatant, add 10 mL ADF Base, and move to 15 mL Falcon for easier pelleting.
  n. Spin at 100 *g* for 3 min at 4°C and remove supernatant (this removes single cells) and add 10 mL ADF Base.
  o. Take 20 μL and count crypts as in step i.
  p. Spin amount to be plated in a 15 mL Falcon at 300 *g* for 3 min at 4°C; aim to plate 500 crypts/well/50 μL MG in a 24-well plate.
  q. Remove supernatant and gently resuspend the pellet in MG (500 μL is enough for ∼12 wells) and plate out on a pre-warmed at 37°C well plate - 50 μL/well.
    Tip: Work swiftly as MG solidifies at room temp and above. Avoid it solidifying and bubbles when mixing with crypts (Figure 2).
  r. Incubate for 10 min at 37°C to allow MG to solidify.
  s. Add 500 μL/well of ADF Org + all growth factors (EGF, R-spondin, Noggin and ChiR) to the side of the well to prevent disrupting the MG dome containing crypts.
  t. Change the media on day 2.
    i. Remove media without disrupting the MG.
    ii. Add 500 μL/well of ADF Org + growth factors (EGF, R-spondin and Noggin).
    iii. Incubate at 37°C.
      **CRITICAL:** Wash all Falcon tubes, tips and serological pipettes briefly in ADF Base to prevent tissue from sticking to plastics.

### Passaging and freezing organoids

**Timing: every 4/5 days**

**Figure 3:**
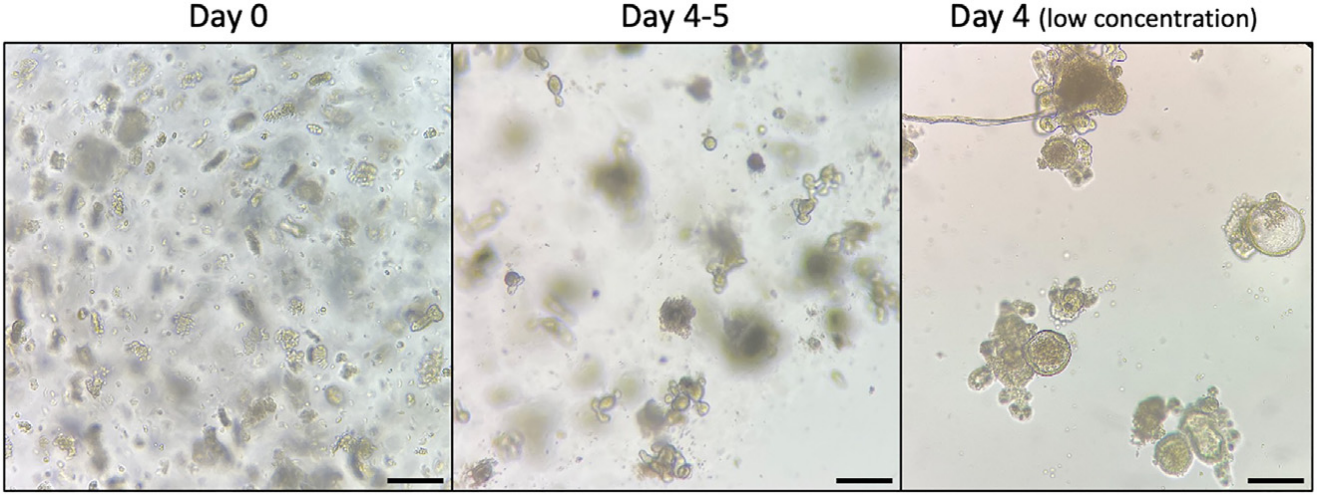
Different stages of the organoid culture Day 0 shows the crypts being cultured in the Matrigel. Days 4–5 show fully grown organoids producing mucus, ready to be split. Day 4 (low concentration) allows better visualization of the shape of the organoids, this concentration is used for stimulation.

Two days after the first media change (at day 2), organoids will be fully differentiated and start shedding mucus/cells, indicating that they are ready to be split and passaged. Organoids will be fully differentiated every 3–4 days when passage is required. See Figure 3 for examples.

3. Passaging Organoids.
  a. [Aspirate media and add 1 mL ice-cold ADF Base.
  b. Break up MG with a 10 mL serological pipette (scrape quite gently).
  c. Combine up to 10 into 15 mL Falcon tube, make up to 10 mL with ice-cold ADF Base, if needed, and spin at 300 *g* for 3 min at 4°C.
  d. Aspirate media, add 1 mL ice cold ADF base and gently break up MG with 1 mL pipette.
  e. Add 10 mL ADF Base and spin at 300 *g* for 3 min at 4°C. Repeat steps 4–5 until all MG is removed.
  f. Aspirate media and use a P200 to disrupt the pellet and break the organoids (×20 gently).
  g. Add 10 mL ADF Base and spin at 100 *g* for 3 min.
  h. Aspirate media and add 10 mL ADF Base. Take 20 μL and count.
    Tip: we want 500 crypts/well/50 μL MG in a 24-well plate.
  i. Spin amount to be plated in a 15 mL Falcon tube at 300 *g* for 3 min at 4°C.
  j. Remove supernatant, add 500 μL MG (enough for ∼12 wells), and plate out 50 μL/well.
    Tip: Work swiftly as MG solidifies at room temp and above. Avoid it solidifying and or allowing bubbles to form when resuspending organoid fragments.
    Tip 2: When plating for induction experiments, using a Nunc Lab-Tek II Chamber Slide System – 8-well chamber slide, use 30 μL MG per well.
  k. Incubate for 10 min at 37°C to allow MG to solidify.
  l. Add crypt media (ADF Org + growth factors (EGF, Noggin, R-Spondin).
  m. Change the media every 2 days.
    i. Remove media without disrupting the MG.
    ii. Add 500 μL/well of ADF Org + growth factors (EGF, Noggin and R-Spondin).
    iii. Incubate at 37°C.
      ***Note:*** The number of crypts per well is very important as too few or too many will affect the growth of the organoids. For stimulation experiments the concentration is lower to improve visibility, 200 crypts/well are enough. Organoids are challenging to establish and maintain. During establishment, it is important to keep all samples on ice when possible and work swiftly. Furthermore, checking the cultures under the microscope every day is essential to keep control of their growth and make sure the passage is performed at optimal times.
4. Freezing Organoids.
  a. Aspirate media and add 1 mL ice cold ADF Base/well.
  b. Break up MG with a P1000 tip (scrape quite gently).
  c. Combine into 15 mL Falcon tube (3 wells of organoids/15 mL).
  d. Spin at 150 *g* for 10 min at 4°C and aspirate media.
  e. Repeat steps 4–5 until all MG is removed.
  f. Resuspend in 1 mL Gibco Cell Recovery freezing media.
  g. Transfer 1 mL to cryovials and freeze at −80°C and put into LN_2_ the following day. A Mr Frosty container (Nalgene) is ideal for overnight freezing in −80°C.

***Note:*** To freeze 1 vial of organoid (1 mL), 3 MG droplets are necessary

5. Thawing Organoids.
  a. Thaw rapidly at 37°C in a water bath.
  b. Spin at 300 *g* for 3 min at 4°C and aspirate media
  c. Add 1 mL of ADF, transfer to a 15 mL Falcon tube and make up the volume to 10 mL with ice-cold ADF.
  d. Spin at 300 *g* for 3 min at 4°C and aspirate media.
  e. Add 500 μL MG (enough for ∼12 wells), and plate out 50 μL/well.
    Tip: Work swiftly as MG solidifies at room temp and above. Avoid it solidifying and or allowing bubbles to form when resuspending pellet containing organoids.
  f. Incubate for 10 min at 37°C to allow MG to solidify.
  g. Add crypt media (ADF Org + growth factors (EGF, Noggin, R-Spondin and, for the initial cultures only, ChiR).
  h. Change the media every 2 days.
    i. Remove media without disrupting the MG.
    ii. Add 500 μL/well of ADF Org + growth factors (EGF, Noggin and R-Spondin).
    iii. Incubate at 37°C.

**Figure 4:**
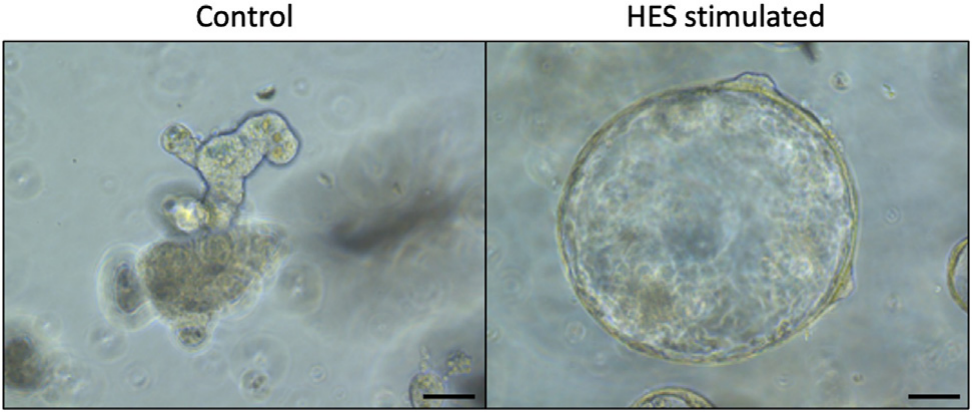
Example of how HES-stimulated organoids look 4 days post stimulation

**Figure 5:**
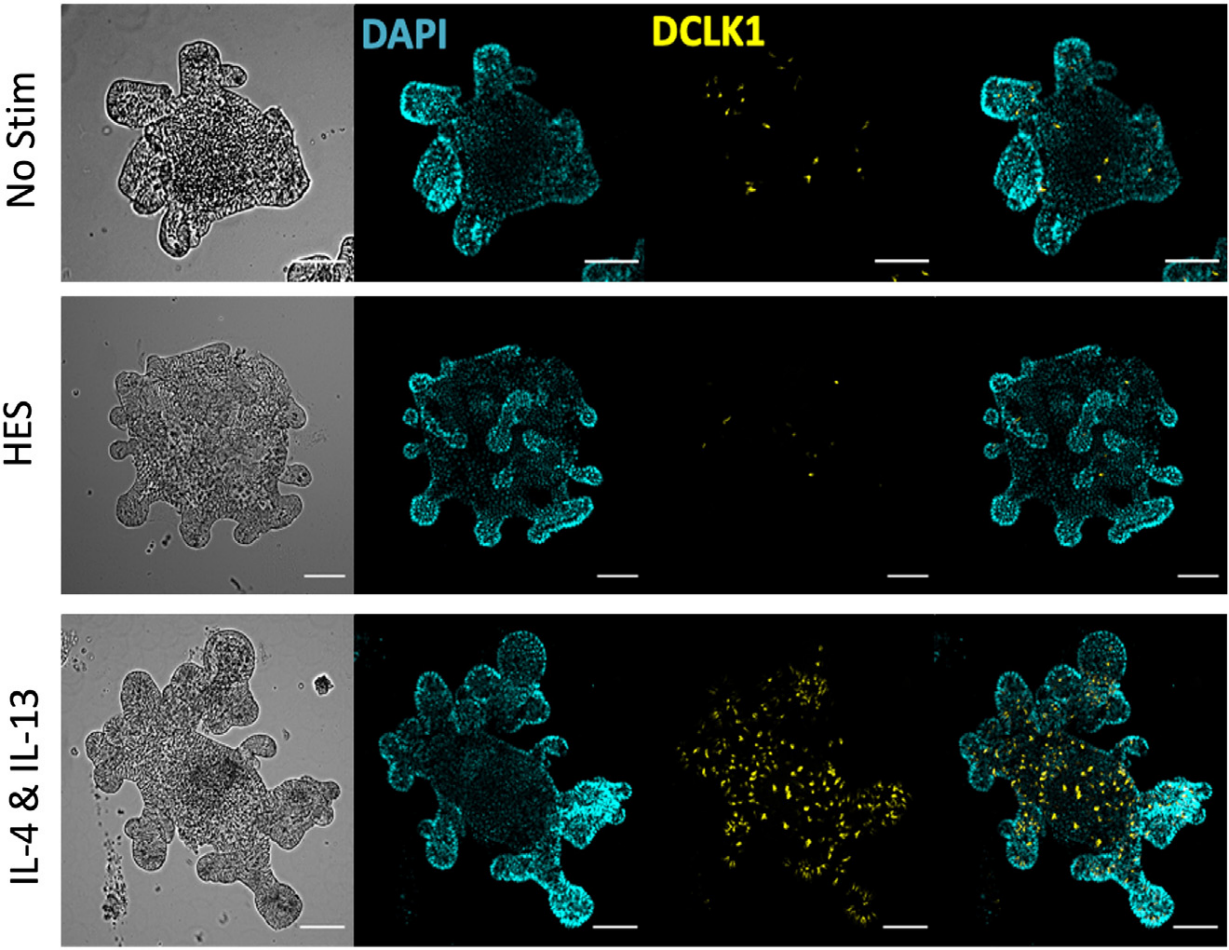
Example of IF stain of stimulated organoids using an anti-DCLK1 antibody for tuft cell stain

### Experimental stimulation of organoids

**Timing: 1–3 days**

Organoids should be split at least four times before being used for experimental treatments such as co-culture with cytokines or parasite products (see Table below). For example, the type 2 cytokines IL-4 and IL-13 can be added, to stimulate preferential differentiation of intestinal stem cells to the goblet cell and tuft cell lineages; conversely, helminth excretory-secretory (ES) products can be administered, which in some cases reverse the effects of these type 2 cytokines.^3^ In this protocol, test agents are added on day 3, but they can be added at any time chosen by the researcher.

6. Day 0 – Stimulate.
  a. Plate fresh organoids in low concentration (200 crypts/well in 30 μL MG) into Nunc Lab-Tek II Chamber Slide System - 8-well chamber slide.
  b. Add 200 μL ADF Org + growth factors (EFG, Noggin, R-Spondin and ChiR) to each well and incubate at 37°C.
7. Day 2 – Change media.
  a. Remove media without disrupting the MG.
  b. Add 200 μL/well of ADF Org + growth factors (EGF, Noggin and R-Spondin).
  c. Incubate at 37°C.
8. Day 3 – Change media and add stimulants.
  a. Remove media without disrupting the MG.
  b. Add 200 mL/well of ADF Org + growth factors (EGF, Noggin and R-Spondin) + stimulants (see Table below for examples).
  c. Take pictures of organoids with an inverted microscope or EVOS imaging system using 4×, 10× and/or 20× objectives.
  d. Incubate at 37°C.

**Table:** 

Stimulant	Concentration	
IFN-γ	1 ng/mL	Type 1 cytokine
IL-4/IL-13	400 ng/mL	Type 2 cytokine
*H. polygyrus* ES	10 μg/mL	Parasitic product
SB431542	10 μM	TGF-β pathway inhibitor

9. Day 4 (24 h post-induction) –.
  a. Take pictures of organoids with an inverted microscope or EVOS imaging system using 4×, 10× and/or 20× objectives.
10. Day 5 – Harvest the cultures for assays as below.

### Analysis of organoids

**Timing: 1–3 days – depends on the method**

Depending on the goal of the experiment this may include (11) Morphology by bright field microscopy; (12) Flow cytometry of dispersed single cells; (13) Immunofluorescence of intact or sectioned organoids; and (14) RNA extraction for qPCR or RNA sequencing.

11. Morphology by bright field microscopy (Figure 4).
  a. Take pictures of organoids with an inverted microscope or EVOS imaging system using 4×, 10× and/or 20× objectives.
  b. Analyze morphological changes using image software such as ImageJ. A technique for measurement and quantification by the area of organoid images has been described by Lindholm et al.^4^
12. Organoid harvest and stain for Flow cytometry.
  a. Harvest organoids by replacing media with 1 mL cold ADF base and scraping with a 10 mL serological pipette. This detaches the MG from the plate into the media.
  b. Pipette out organoids in MG/media into a 15 mL falcon and top up to 10 mL with cold ADF base. Spin at 300 *g* for 4 min.
  c. Aspirate off the media and use a P200 to disrupt the pellet (×10, gently).
  d. Add 0.5–1 mL TrypLE express and 2000 units/mL DNase II. For 3 wells of organoids, 0.75 mL, for 2, 0.5 mL.
  e. Leave at 37°C for 20–25 min to dissociate organoids. Place in the 37°C incubator shaker, as shaking should encourage dissociation.
  f. At the end of incubation, inactivate TrypLE by adding 5 mL of ADF base with 5% FCS.
  g. Pipette the supernatant 5–10× to dissociate the organoids. They may be visible as distinct organoids before pipetting, after which they present a cloudy cell suspension.
  h. Pass the cell suspension through at 40 μm cell strainer.
  i. Count using trypan blue. Calculate the amount in hand and plate out appropriately for flow. Ideally a million cells for each well should be used, however organoids do not yield this amount. For this reason, 20 × 10^4^ cells/well can be used.
  j. Spin down 400 *g* for 5 min the correct amount (usually all that is available) and resuspend in the correct amount of PBS for plating out 100 μL/ well. Plate into a 96-well round-bottomed plate.
  k. Spin down at 400 *g* for 5 min at 4°C and wash once more in PBS (100 μL) to get rid of any trace of FBS.
  l. Incubate with live/dead dye for 30 min at 4°C in the dark (BV506, 1:1000 dilution, use 100 μL /well).
  m. Spin down at 400 *g* for 5 min, then wash with 200 μL PBS.
  n. Block using rat IgG (1:50, 50 μL per well) for 20 min at 4°C.
  o. Wash using 150 μL FACS buffer (add to the 50 μL in the well, then spin at 400 *g* for 5 min).
  p. Stain in 50 μL of extracellular stain mix, usually these antibodies are used at a 1:200 dilution. Can stain in FACS buffer, or may need Brilliant Stain Buffer if using 2 or more BD Brilliant dyes). Leave for 35 min at 4°C in the dark.
  q. Add 150 μL PBS to wash and spin at 400 *g* for 5 min.
  r. Resuspend in 50 μL PBS, then fix cells by adding 100 μL of 4% PFA. Leave at room temperature for 15 min.
  s. Spin down, then wash once with 200 μL PBS.
  t. Permeabilize cells in 150 μL PBS 1% Triton X-100 for 15 min at room temperature.
  u. Stain in 50 μL of intracellular antibodies suspended in PBS 1% BSA. Leave in the dark at room temperature for 1 h.
  v. If using secondary antibodies for the intracellular stain, wash with 150 μL perm buffer.
  w. Incubate in 50 μL secondary antibodies, if needed. Leave in the dark for 30 min at room temperature.
  x. Wash twice in 200 μL FACS buffer. The cells can be left in FACS buffer until ready to flow.
  y. Analyze the cell suspension with a flow cytometer (or use a cell sorter to isolate a specific cell population for biological assays).

***Note:*** selection of the fluorescent Ab should be made according to your flow machine. Some examples of Abs we use are on the reagent list.

13. Indirect Immunofluorescent Staining of Intestinal Organoids (Figure 5).
  a. For fixation, aspirate the medium from each well, wash 2× in cold PBS and immediately fix with 4% paraformaldehyde for 20 min at room temperature.
    Tip: Once fixed, slides can be stored at 4°C for up to two weeks. Rinse wells once in PBS and store in 500 μL/well PBS. Wrap wells with parafilm to avoid drying out.
  b. Permeabilize with PBS containing 0.5% Triton X-100 for 10 min at 4°C. Depending on the antibody utilized for immunostaining, the detergent concentration or duration of permeabilization may require modification.
  c. Rinse 3 times with PBS/Glycine (1× PBS; 100 mM glycine), 10–15 min per wash at room temperature.
  d. Incubate with 200 μL/well IF buffer for 45–60 min at room temperature.
  e. Primary antibody: Incubate with the primary antibody in Antibody Dilution Buffer overnight (15–18 h) at room temperature.
  f. Rinse 3 times (5–10 min each) with IF Buffer at room temperature with gentle rocking.
  g. Incubate with fluorescent conjugated secondary antibody in Antibody Dilution Buffer for 40–50 min at room temperature.
  h. Rinse once (5 min) with IF Buffer at room temperature with gentle rocking.
  i. Rinse 2–3 times with PBS (5 min) at room temperature.
  j. Remove chambers from slides.
  k. Add nuclear stain (DAPI 1:10,000 in water for 10 min).
  l. Rinse 2–3 times with PBS (5 min) at room temperature.
  m. Mount with VECTASHIELD.
14. RNA extraction for qPCR.
  a. Aspirate medium from each well.
  b. Wash wells with 400 μL ice-cold PBS.
  c. Add 400 μL Trizol to each well, in the fume hood.
  d. Incubate on ice for 10 min, to help MG dissolve.
  e. Pipette up and down to completely dissolve MG and transfer to an Eppendorf tube.
  f. Vortex for 10 s.
     → Store at −80°C until ready to continue with RNA extraction.
  g. Centrifuge at 12,000 *g* for 2 min at 4°C and transfer supernatant to a new Eppendorf tube to remove debris.
  h. Add 100 μL chloroform to the supernatant before mixing well and incubate at room temperature for 3 min.
  i. Centrifuge at 12,000 *g* for 15 min at 4°C.
  j. Transfer the upper aqueous phase to a new Eppendorf tube.
    Important: Make sure not to transfer any material of the interphase or organic phase to avoid phenol/protein/DNA contamination.
  k. Add 1.5 volumes of supernatant, of 100% ethanol to precipitate RNA. Mix well by inverting the tube several times.
  l. Proceed to RNA purification using RNeasy Mini kit following manufacturer’s instructions.
    ***Note:*** It is important to remember that more than one well may be needed for flow and RNA extraction experiments to provide a sufficient number of cells per sample.

## Expected outcomes

This protocol will allow you to keep a small intestine organoid culture over time, including the generation of stocks for future experiments.

The different analysis methods will provide a great variety of results allowing the researchers to gain great insight on different levels post stimulations. From morphological changes to RNA expression including cell numbers and their location within the organoid.

## Limitations

• These organoids are formed purely of epithelial cells. Therefore, the contributions of stromal and immune cells are not included in these experiments.
• Along this topic, gut organoids lack some specialized intestinal epithelial structures, specifically Peyer’s patches and their M (microfold) cells.
• The luminal surface is inaccessible in growing organoids unless microinjection is used. Although 2D techniques are available the cultures are very short-lived.^5^

## Troubleshooting

• If organoids do not grow well or are growing slowly, check the concentration of your ADF org and the other supplements. If after one media change, they do not recover, try splitting them and increasing the number of organoids per well.
• If organoids fail to bud, check your MG is not heavily diluted on media. As some media may be left on the falcon tube where you have isolated the organoids, it is important to ensure the volume of MG added is about 5 times more than the volume of media left on your tube to ensure its solidification. Ensure 10 min have passed for the domes to form before adding media. Media shouldn’t be added cold, room temperature is better to conserve MG integrity.
• It is better to optimize all IF antibody dilutions in tissue sections prior to staining organoid slides, to avoid problems with samples that have taken considerable time to generate.

## Resource availability

### Lead contact

Further information and requests for resources and reagents should be directed to and will be fulfilled by the lead contact, Rick Maizels (Rick.Maizels@glasgow.ac.uk).

### Materials availability

This study did not generate new unique reagents.

## Data Availability

This study did not generate/analyze datasets/code.
